# Synergistic Changes in Ascorbic Acid and Soluble Sugar Contents in Apricot Fruits and the Related Regulatory Mechanisms

**DOI:** 10.1002/fsn3.70899

**Published:** 2025-09-09

**Authors:** Yanhong He, Yang Zhao, Tianxing Guo, Wenbao Jia, Chun Tian, Yu‐e Bai

**Affiliations:** ^1^ Academy of Forestry Inner Mongolia Agricultural University Hohhot China; ^2^ Wuhai City Natural Resources Development Center WuHai China; ^3^ Inner Mongolia Autonomous Region Hospital of Traditional Chinese Medicine Hohhot China

**Keywords:** apricot, ascorbic acid content, fruit developmental stage, soluble sugar content, transcriptome sequencing, WGCNA

## Abstract

Soluble sugars, including sucrose, are vital to the overall quality of apricot fruits, influencing attributes such as sweetness, flavor, and texture. As the primary soluble sugar, sucrose plays a central role in determining fruit quality. The synthesis and accumulation of sucrose, as well as its interaction with ascorbic acid metabolism, are regulated by various enzymes, including sucrose synthase and invertase. While several genes related to sucrose metabolism have been identified in apricots, the mechanisms by which sucrose and ascorbic acid work together to regulate fruit quality remain “is not fully elucidated”. In the present investigation, the changes in soluble sugar and ascorbic acid levels throughout fruit development were examined, and the mechanisms underlying the changes in soluble sugar and ascorbic acid levels were examined in ‘Wuyuexian’ apricots (WYX) and ‘Lanzhoudajie’ apricots (LZ). The data revealed that the sucrose content of ‘WYX’ was significantly higher than that of ‘LZ’, whereas the sorbitol levels of ‘LZ’ were higher than ‘WYX’. The fructose contents of both varieties increased later in growth, and the ascorbic acid content of ‘LZ’ was three times higher than that of ‘WYX’ during the early stage of fruit growth. These trends became intensified in both varieties after the rapid growth stage, with ‘WYX’ having the highest sucrose content. Additionally, transcriptomic analysis, K‐means analysis, and weighted correlation network analysis (WGCNA) howed the expression levels of genes related to sucrose and ascorbic acid metabolism, the synergistic effects of gene clusters, and the complex associations among genes. Moreover, the key hub genes *HSFB1* and *FTIP4* were identified, which play crucial roles in regulating sucrose and ascorbic acid metabolis. However, transcriptome analysis of gene expression only partially reflects metabolism regulation, and further in‐depth investigations are required to elucidate other regulatory mechanisms. Our research revealed a relationship between the synthesis of ascorbic acid and soluble sugars in apricots and offers new perspectives on molecular breeding and germplasm development in apricots and establishment of germplasm resources.

## Graphical abstract

This study investigates the dynamic changes in soluble sugar and ascorbic acid contents during apricot fruit development, focusing on two varieties, ‘Wuyuexian’ (WYX) and ‘Lanzhoudajie’ (LZ). By combining transcriptomic analysis and metabolic profiling, the research uncovers the regulatory mechanisms behind sugar and ascorbic acid synthesis, identifying key genes such as HSFB1 and FTIP4 that play pivotal roles in metabolic processes. The findings reveal significant differences in sugar accumulation and ascorbic acid content between the varieties, with WYX showing higher sucrose levels and LZ exhibiting higher ascorbic acid during early growth stages. This work provides valuable insights into apricot fruit quality enhancement and lays the foundation for future breeding programs focused on improving nutritional content and fruit quality.

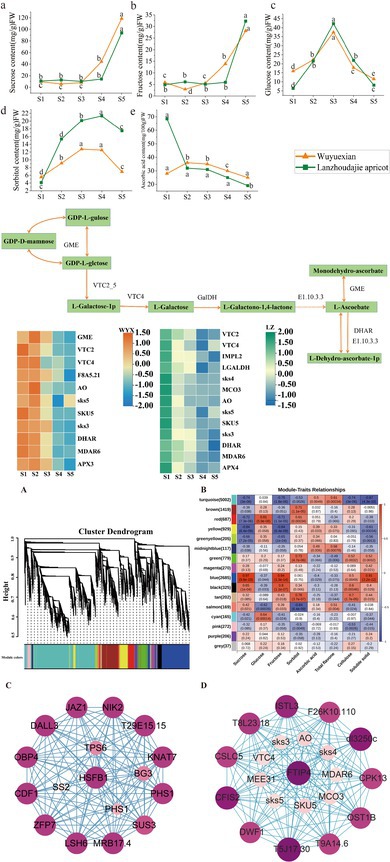

**Abbreviations**
LZ: ‘Lanzhoudajie’ apricots
WYX: ‘Wuyuexian’ apricots

## 1 Background

The apricot (*Prunus armeniaca L*.) is an important species belonging to the Rosaceae family and the “genus Prunus”. It is cultivated throughout the Mediterranean region, South Africa, South America, and North America and is a popular seasonal fruit that people enjoy eating worldwide. It is the third most extensively grown drupe fruit tree in the world (Alajil et al. 2021; Fan et al. 2017). Apricots were originally domesticated in China, and their cultivation can be traced back 2600 years (Karatas 2022). In addition to their wonderful flavor, appealing aroma, and vibrant color, apricot fruits are also packed with minerals, including ascorbic acid, β‐carotene, and phenolics, which are valued for their nutritional and economic benefits for their nutritional and economic value (Zhou et al. 2020; Jiang et al. 2019; Crouzet et al. 1990).

As one of the essential components of plant metabolism, sucrose serves as a key component for n plant growth and development (Ackermann et al. 1992; Kroger et al. 2006; Pangborn 2006; Ren et al. 2018; Lingle and Dunlap 1987). Sucrose synthase (SUS), which regulates the synthesis of UDP‐glucose, is primarily responsible for controlling the synthesis of sucrose in plants (Koch 2004). The level of the rate‐limiting enzyme in sucrose synthesis and the enzyme that breaks down sucrose, sucrose phosphate synthase (SPS), is positively correlated with the accumulation of sucrose (Wei et al. 2020; Su et al. 2021). Invertases convert sucrose to fructose and glucose. Furthermore, fructokinase and hexokinase can phosphorylate glucose and fructose to produce glucose‐6‐phosphate and fructose‐6‐phosphate (Rolland et al. 2006).

Recent studies, high‐throughput sequencing and biotechnological techniques have revealed numerous enzyme‐encoding genes linked to sucrose synthesis and metabolism in apricot fruits (Aslam et al. 2019; Zhang et al. 2019). During the total sugar and sucrose accumulation stages of apricot fruit development, the expression of sucrose synthase genes *PaSUS1*, *PaSUS3*, *ParSuSy5*, *ParSuSy6*, and *ParSuSy7* was markedly upregulated in response to high sucrose concentrations. It was suggested that these genes are involved in the synthesis of proteins by sucrose synthase (Iqbal et al. 2020). Lombardo et al. (Lombardo et al. 2011) reported that after fruit discoloration, the transcript levels of sucrose synthase and sucrose phosphate synthase dramatically increased, but the transcript levels of sorbitol dehydrogenase did not significantly change over time. Wang et al. (Wang et al. 2019) reported a correlation between the sugar content of ripe apple fruits and the expression of the apple hexose transporter protein MdHT2.2, whose primary function is to uptake and move glucose and fructose from the cell wall space into cells, which helps maintain the ability to cells to release hexose.

Ascorbic acid (AsA) is abundant in apricot fruit and is one of the most widely distributed water‐soluble antioxidants found in both plants and animals. As an enzyme cofactor, scavenger of free radicals, and electron donor and acceptor in electron transport across membranes or chloroplasts, AsA is essential for life. It plays important roles in various plant processes, including growth and development, hormone signaling, cell division and proliferation, and reactive oxygen species scavenging. As a result, it protects DNA, proteins, and lipids from oxidative damage during photosynthesis and under abiotic stress (Davey et al. 2000; Mellidou and Kanellis 2017; Fenech et al. 2018). The pathways involved in the synthesis of D‐mannose/L‐galactose (D‐Man/L‐Gal), D‐galacturonic acid (D‐GalA), L‐Gulose, and inositol (Myo‐inositol) are also involved in the production of ascorbic acid in plant fruits (Paciolla et al. 2019). Additionally, although ascorbic acid is an essential nutrient, the body is unable to synthesize ascorbic acid and must instead obtain it from external sources. As the apricot fruit develops, its ascorbic acid level changes from high to low (Li 2013). Research on genetic and biochemical techniques for plant regulation, particularly for ascorbic acid biosynthesis, is relatively limited, and the enzymes involved in ascorbic acid biosynthesis remain unidentified (Yin et al. 2024).

In this study, the ascorbic acid and soluble sugar contents of two varieties of apricot fruits at five developmental stages were examined. Although significant progress has been made in understanding the individual roles of sugars and ascorbic acid in fruit development, there remains a critical knowledge gap regarding their synergistic regulation. This gap is particularly evident in apricots, where the combined influence of sugars and ascorbic acid on fruit quality has not been fully explored. This study aims to address this gap by investigating the interactions between sugars and ascorbic acid throughout fruit development. Furthermore, we employed transcriptome analysis, weighted correlation network analysis (WGCNA), and RT–qPCR to identify the major genes involved in the synthesis of soluble sugars and ascorbic acid in apricots. This study will advance of artificial breeding and germplasm resources and enhance the nutritional value and overall quality of apricots.

## 2 Methods

### 2.1 Plant Materials

The apricot grove in the Wanjiagou Autonomous Region of Inner Mongolia, Tuzuo Banner, Hohhot, was the source of the Wuyuexian apricot ‘WYX’ and Lanzhoudajie apricots ‘LZ’. Fruiting started on the sixteenth day after flowering, and sampling was performed every seven days after fruiting. For each variety, three apricot trees with consistent growth conditions were chosen. Undamaged fruits of similar size and appearance were chosen for sampling in each stage. The samples were sliced quickly into uniformly small pieces, snap‐frozen in liquid nitrogen, covered with tin foil, and stored at −80°C. Every sample had three biological replicates.

### 2.2 Detection of Glucose, Sucrose, Fructose, and Sorbitol Contents

Under the action of specific enzymes, sucrose is converted into glucose and fructose, and glucose, through the enzyme complex including hexokinase, reduces NADP+ to NADPH. By measuring the increase in NADPH at 340 nm, the contents of sucrose, glucose, and fructose can be determined separately (GERUISI, China, G0560F).

Tissue Sample: Weigh approximately 0.1 g of tissue, add 1 mL of distilled water, and homogenize on ice. Centrifuge at 12,000 rpm at room temperature for 10 min. Add reagents as instructed in the manual for detection. All steps are performed strictly according to the manual.

### 2.3 Determination of Ascorbic Acid Content

Reduced ascorbic acid (AsA) can reduce ferric ions (Fe^3+^) to ferrous ions (Fe^2+^), and the ferrous ions react with red phenantroline to form a red complex. This complex has a characteristic absorption peak at 534 nm, and its intensity is directly proportional to the content of reduced ascorbic acid (GERUISI, China, G0201F).

Tissue Sample Treatment: Weigh approximately 0.1 g of tissue, add 1 mL of pre‐cooled extraction solution, and homogenize on ice. After extracting at room temperature for 10 min, centrifuge at 12,000 rpm, 4°C for 10 min. Add reagents as instructed in the manual for detection. All steps are performed strictly according to the manual.

### 2.4 RNA Extraction, RNA‐Seq and Sequence Assembly

Twenty‐four libraries were created via RNA extraction using a Tiangen polysaccharide polyphenol kit (TIANGEN, Beijing). Three biological replicates of each sample were included in the analysis. Gel electrophoresis (voltage 180 V for 16 min) using 1% agarose gel and an Agilent 2100 Bioanalyzer was performed to test the purity and integrity of the extracted RNA. RNA purity was measured using a NanoPhotometer (OD260/280 and OD260/230). NA concentration was measured using Qubit 4.0 fluorescence. RNA integrity was assessed using a Qsep400 Bioanalyzer.

Following total RNA extraction, mRNA was enriched using Oligo (dT) magnetic beads and fragmented with a fragmentation buffer. Short segments of mRNA were used as templates to synthesize first‐strand cDNA. The Cdna was then purified by adding buffer, dNTPs, and DNA polymerase I to create double‐stranded cDNA. Finally, the purified cDNA was with a poly‐A tail and end repair for sequencing. These processes in addition to sequence splicing, fragment size selection using AMPure XP beads, and PCR were the methods used to construct the cDNA library. After the insert size satisfied expectations, the synthesis of the cDNA library was completed by quantifying the library concentration. The Illumina HiSeq platform was then used to sequence the mixed samples. RNA‐seq analysis was conducted with three biological replicates, and differential gene expression was identified using the DESeq2 package. False Discovery Rate (FDR) correction was applied, with a significance threshold set at *p* < 0.05.

StringTie 2.1.6 assembly software was used to splice the raw data after they had been filtered, the sequencing error rate and GC content distribution were examined, and clean reads were obtained. After hierarchical clustering by Corset (official website: https://code.google.com/p/corset‐project/), the spliced transcript sequences were inputted into the UniGene database to obtain the longest UniGene cluster sequences for further analysis.

### 2.5 Expression Quantification and Screening of Differentially Expressed Genes (DEGs)

The acquired sequence number was normalized to the FPKM value; gene expression levels were determined by normalizing the FPKM value. Using the DESeq2 plug‐in of TBtools and the negative binomial distribution model, DEGs were identified. The following screening parameters were used to identify DEGs in the mature apricot fruits: FDR < 0.05 and |log2Fold Change| ≥ 1.

### 2.6 Functional Annotation of UniGene Clusters

After the amino acid sequences of the UniGene clusters were predicted, sequence comparison was carried out using the Pfam database and HMMER software to obtain the functional annotation information of the corresponding UniGene clusters. UniGene cluster sequences were compared using the Kyoto Encyclopedia of Genes and Genomes (KEGG), NCBI nonredundant (NR), Swiss‐Prot, Gene Ontology (GO), Eukaryotic Orthologous Groups (KOG), and Trembl databases via BLAST software (*E* value ≤ 10^−5^).

### 2.7 Coexpression Network Construction and Identification of Hub Genes

The DEGs identified in 30 samples of ‘WYX’ and ‘LZ’ were subjected to WGCNA. After initial DEG filtering, the samples were subjected to WGCNA with a soft threshold of β‐value = 14, a minimum module gene number of 50, and a similar module merging threshold of 0.25. The IQR filter threshold was 0.5. To identify key transcription factors in metabolic pathways, the target module gene coexpression network was visualized using maximum clique centrality (MCC) topology analysis, with a degree value ≥ 1.

### 2.8 Validation of RNA‐Seq Data via RT–qPCR

To confirm the accuracy of the RNA‐seq data, RT–qPCR was performed to measure the expression levels of the 11 DEGs in the two apricot varieties. As described in section 2.4.1, RNA was extracted and utilized as a template. Next, DNA was produced via reverse transcription using the PrimeScript FAST RT reagent kit with gDNA Eraser, and spare reagents were stored in a refrigerator at −20°C.

RT–qPCR amplification was carried out using TB Green Premix Ex TaqTM II (Tli RNaseH Plus) and a Light Cycler 480 device. The total volume of the reaction mixture was 20 μL and was composed of 10 μL of TB Green Premix Ex Taq II (Tli RNaseH Plus) (2X), 1 μL of sample cDNA, 1 μL of each of the upstream and downstream primers (20 μmol/L), and 7 μL of ddH2O. The reaction procedure was the following: predenaturation (95°C, 30 s, 1 cycle); amplification (95°C, 5 s; 60°C, 30 s, 40 cycles); melting (95°C, 5 s; 60°C, 1 min; 95°C, 1 cycle); cooling (50°C, 30 s, 1 cycle); and storage at 4°C. Primer5 was utilized in the construction of the primers, and the internal reference gene was the 18S gene from the almond genome (Lin 2023). Four technical duplicates were set up. For qRT‐PCR, each gene was measured in three biological replicates, and data were analyzed using the 2^−ΔΔCt^ method to ensure the statistical reliability of the results (Livak and Schmittgen 2001).

### 2.9 Data Analysis

SIMCA14.1 software was employed to analyze the principal component analysis (PCA) results; Origin Pro 2021 software was used to analyze data and generate Venn diagrams, histograms, differentially abundant metabolite heatmaps, KEGG bubble plots, and hierarchical cluster diagrams. Metabolic pathway diagrams were generated using PowerPoint and Adobe Illustrator CC 2017SP. GraphPad Prism 8 v8.0.2, TBtools, and Adobe Illustrator 2020 were used to create heatmaps depicting the expression of DEGs. The WGCNA package of R language software was used to generate and analyze weighted gene coexpression networks for DEGs, and Cytoscape 3.9.1 was employed to visualize the gene interactions within the target module.

## 3 Results

### 3.1 Soluble Sugar Content in the Two Apricot Varieties During Fruit Development

In this experiment, the changes in the sugar contents of ‘WYX’ and ‘LZ’ across five stages of fruit growth (S1: young fruit stage; S2: fast‐growing stage; S3: nodule stage; S4: color‐transitioning stage; and S5: commercial ripening stage) were analyzed.

**FIGURE 1:**
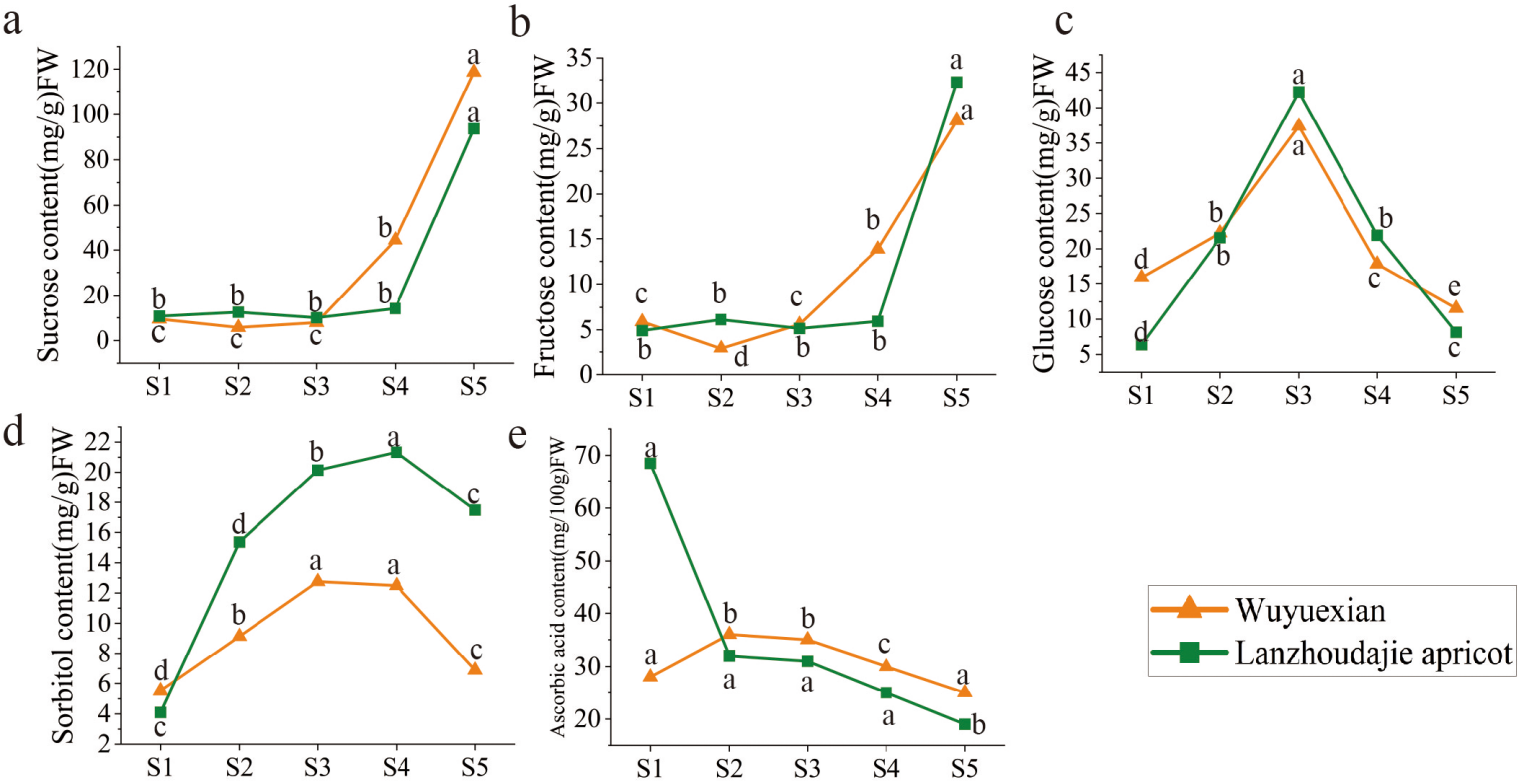
Soluble sugar and ascorbic acid contents of apricot fruits at different developmental stages: (a) Sucrose, (b) sorbitol, (c) glucose, (d) fructose, (e) ascorbic acid.

The sucrose content of ‘WYX’ increased significantly throughout the five periods (Figure 1a), peaking at 44.45 and 118.73 mg/g in S4 and S5, respectively. These findings suggest that the final developmental stages of ‘WYX’ are characterized by a notable increase in sucrose accumulation. Moreover, the sucrose content of ‘LZ’ also increased during the five periods, except for a brief decrease in S4, when it peaked at 93.76 mg/g. The sucrose levels of ‘WYX’ were much greater than those of ‘LZ’ in the late stages, particularly in S5. Moreover, the increase in the sucrose content of ‘LZ’ in S5 was not significant overall. Finally, ‘WYX’ had a sucrose content that was almost 25% greater than that of ‘LZ’.

From S1 to S3, the sorbitol content of ‘WYX’ increased progressively and peaked at 12.76 mg/g (Figure 1b) before declining in S4 and S5. This pattern revealed that sorbitol was required for the growth of ‘WYX’ in the early stages. The sorbitol content of ‘LZ’ increased progressively throughout the five periods, peaking at S4 and S5, where it reached 21.33 and 17.49 mg/g, respectively. These findings indicated that more sorbitol accumulated in the late stage of ‘LZ’ growth than in the other stages. Particularly in S4 and S5, sorbitol levels were much greater in ‘LZ’ than in ‘WYX’, and in S4, the sorbitol concentration in ‘LZ’ was almost 70% greater than that in ‘WYX’.

The glucose content of ‘WYX’ increased significantly between S1 and S3, peaking at 37.40 mg/g (Figure 1c). Then, it stabilized in S4 and S5. The glucose content of ‘LZ’ also increased significantly from S1 to S3, reaching a peak of 42.25 mg/g before beginning to decrease in S4 and S5. Although ‘LZ’ had a greater glucose content in S3 than ‘WYX’, the general changes were similar. Glucose accumulation in both varieties peaked at S3, and the glucose content of ‘LZ’ was nearly 13% higher than that of ‘WYX’.

Throughout the five stages, the ‘WYX’ fructose content progressively increased, reaching 13.86 and 28.08 mg/g in X4 and X5, respectively (Figure 1d). These findings suggested that fructose levels significantly increased in the later stages of ‘WYX’ development, which was related to its role in different stages of growth and development. Throughout the five periods, the ‘LZ’ fructose level varied very little, peaking at 32.26 mg/g in X5. During the S5 period, the ‘LZ’ fructose content increased, but overall, the concentration was quite constant.

The ascorbic acid concentrations of ‘WYX’ were 28, 36, 35, 30, and 25 mg/100 g throughout the five periods (Figure 1e). Overall, during S2, the ascorbic acid concentration peaked at 36 mg/100 g and then progressively decreased. This finding suggested that ascorbic acid synthesis occurred more in the earlier stages of growth in ‘WYX’ than in the later stages. Throughout the five periods, the ‘LZ’ ascorbic acid concentration varied less: it was 68.5, 32, 31, 25, and 19 mg/100 g in the five stages. During S1, the amount of ascorbic acid reached a maximum value of 68.5 mg/100g and subsequently steadily decreased. This finding suggested that although ascorbic acid production in ‘LZ’ was lower in the latter phases, it was still much higher than that in ‘WYX’ in the early stages. During S1, the ‘LZ’ ascorbic acid level was approximately 145% higher than that of ‘WYX’.

### 3.2 Transcriptome Sequencing and Gene Expression Analysis of Two Apricot Varieties in Different Developmental Stages

Thirty samples from the two apricot varieties were sequenced at five critical stages to investigate the mechanism of soluble sugar and ascorbic acid accumulation in apricot fruits. A total of 214.65 Gb of clean data were acquired; each sample's clean data exceeded 6 Gb, and 93% or more of the Q30 bases were present. HISAT was used to compare the clean reads to the *Prunus armeniaca* F106 reference genome in the Genome Database for Rosaceae (GDR). Thirty libraries were sequenced from the clean reads, and the results revealed purities of 91.40% to 95.39% and 95.37% to 98.37%.

**FIGURE 2:**
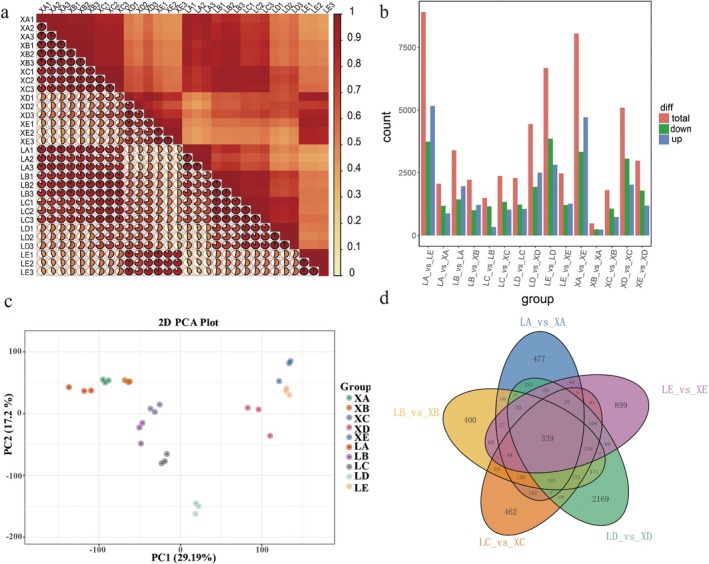
Gene expression correlation in two apricot varieties at five stages. (a) The higher the correlation between two duplicate samples is, the closer the absolute value of the Pearson correlation coefficient (*r*) is to 1. (b) PCA plots illustrating the transcriptome clustering for each of the five developmental stages. (c) The five developmental stages of ‘WYX’ and ‘LZ’ are shown in horizontal coordinates, whereas the total numbers of upregulated and downregulated genes between and within the varieties are represented in vertical coordinates. (d) Venn diagrams illustrating the unique and shared genes between ‘WYX’ and ‘LZ’ in five developmental stages.

On the basis of the FPKM values of the sample genes, transcriptome data were utilized to perform a PCA of the 30 samples; the statistical findings are displayed in Figure 2a. A relatively significant correlation was found between biological duplicates of the same set of samples, indicating strong replicability among the chosen samples. The five growth periods of the two apricot varieties were roughly divided into two groups according to the results of the PCA (Figure 2b). The first three developmental stages (XA, XB, XC, LA, LB, and LC) of ‘WYX’ and ‘LZ’ were in one group, and there was a greater correlation between these stages. The commercial ripening stages of ‘WYX’ and ‘LZ’ were in the other group, and ‘LZ’ color change stage was in its own group.

The genes expressed in the two varieties during the five developmental stages were identified using the criteria of |log_2_Fold Change| ≥ 1 and *p* < 0.05 to determine the genes involved in soluble sugar and ascorbic acid synthesis, as shown in Figure 2c. As shown in Figure 2b, 2063, 2224, 2371, 4444, and 2477 DEGs were differentially expressed among XA vs. LA, XB vs. LB, XC vs. LC, XD vs. LD, and XE vs. LE, respectively. Among these genes, 882/1181 (XA vs. LA), 1219/1005 (XB vs. LB), 1029/1342 (XC vs. LC), 2504/1940 (XD vs. LD), and 1265/1212 (XE vs. LE) genes were significantly differentially expressed in the apricots. A Venn diagram was used to analyze the data further, as shown in Figure 2d. Among the 339 shared DEGs, several DEGs were found to be shared between the two apricot varieties in each of the five growth periods (LA vs. XA (477), LB vs. XB (400), LC vs. XC (462), LD vs. XD (2169), and LE vs. XE (899)).

### 3.3 Dynamic Analysis of Transcriptome Data

**FIGURE 3:**
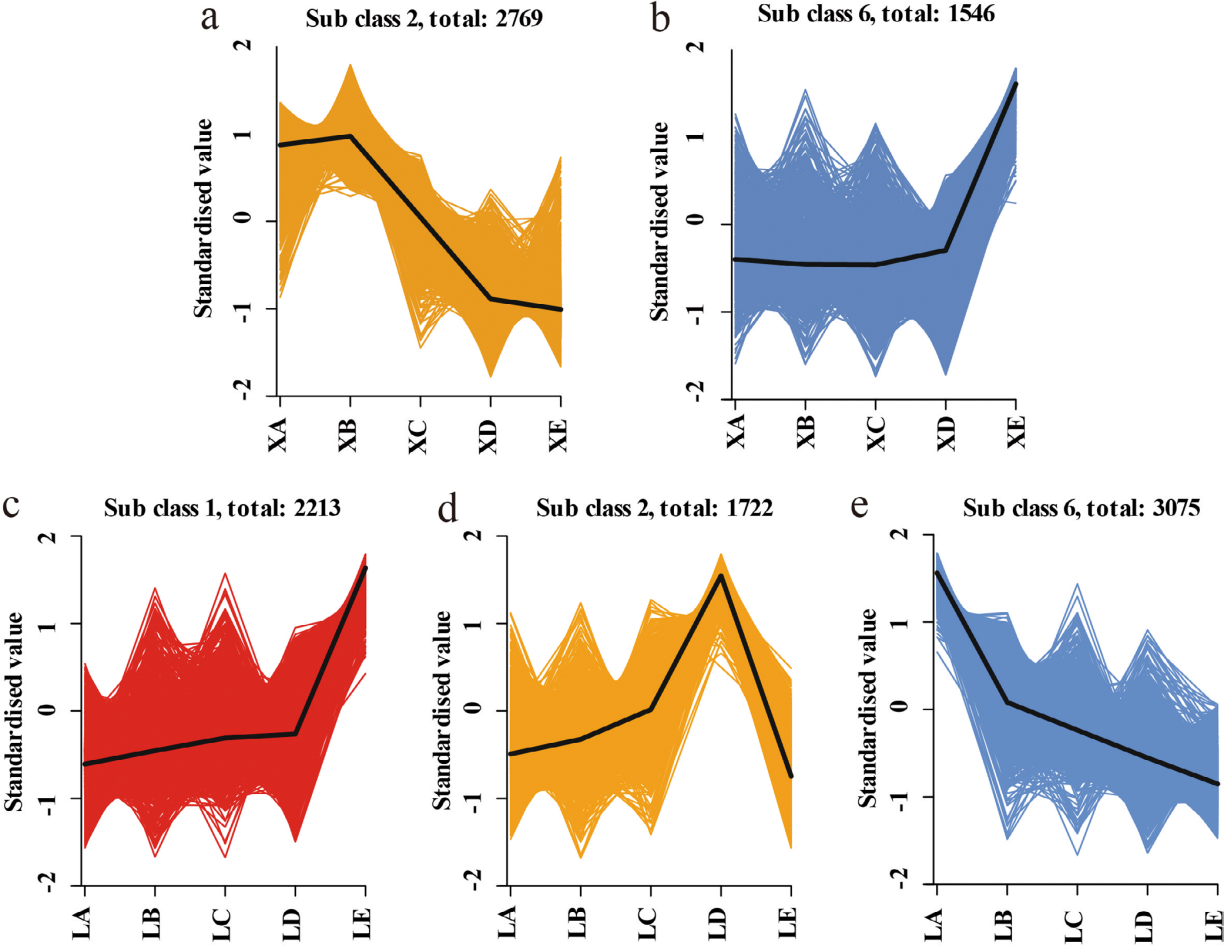
Cluster plot of DEGs using *K*–means algorithm for apricot gene expression (a) X2 (b) X6 (c) L1 (d) L2 (e) L6.

Using the scale function of the R package, the FPKMs of the DEGs were normalized to further investigate the expression of DEGs in the two apricot varieties at various stages of growth and development (Figure 3). A K‐means (k‐means clustering algorithm) clustering analysis was subsequently conducted. DEGs with similar functions presented comparable patterns of change in different developmental periods. There were 13,411 and 13,442 DEGs in ‘WYX’ and ‘LZ’, respectively. These genes were grouped into 10 distinct clusters. On the basis of the trends in soluble sugar and ascorbic acid concentrations, we were able to determine the clustering profiles (‘WYX’: Class2; ‘LZ’: Class6) that corresponded to the trends in sugar metabolism (‘WYX’: Class6; ‘LZ’: Class1) and DEGs involved in ascorbic acid synthesis. According to the results, there were 1546 DEGs in ‘WYX’ and 2213 DEGs in ‘LZ’ involved in sugar metabolism; in the class corresponding to ascorbic acid, there were 2769 DEGs in ‘WYX’ and 3075 DEGs in ‘LZ’; additionally, in the sugar metabolism clusters, the phenotypic changes in ‘WYX’: Class 2 and ‘LZ’: Class 2 were opposite of each other. Interestingly, the trends in ‘WYX’ glucose metabolism and ascorbic acid metabolism were similar.

### 3.4 KEGG and GO Analyses Based on K‐Mean Clustering

**FIGURE 4:**
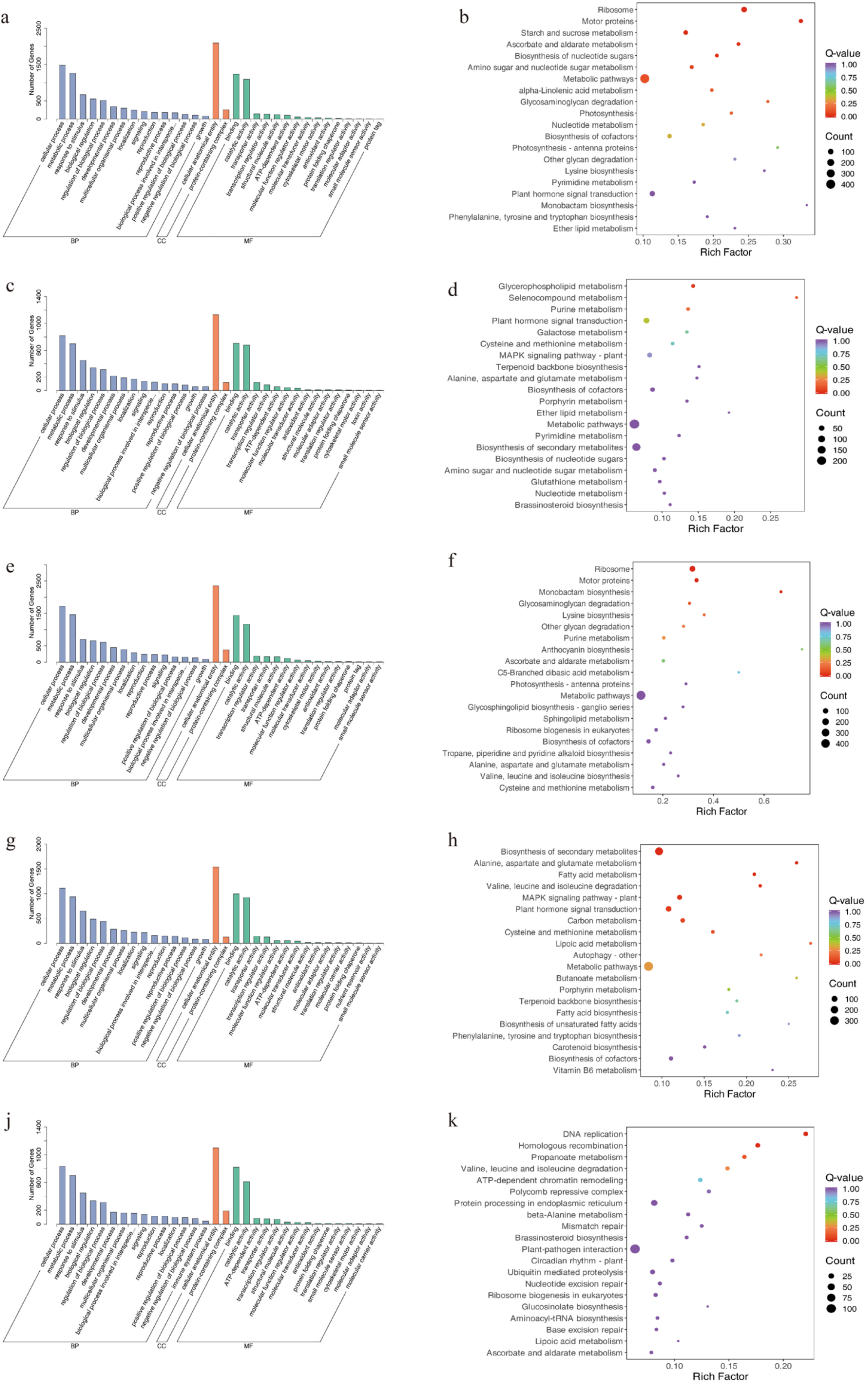
Bar graphs of GO enrichment and scatter plots of KEGG. (a) X2'GO; (b) X2′KEGG; (c) X6′GO (d) X6′: KEGG (e) L1′GO (f) L1′KEGG (g) L2′GO; (h) L2′KEGG; (i) X6′GO (j) L6′KEGG.

The top 15 and top 2 DEGs in ‘WYX’ and ‘LZ’ were chosen for GO biological process (BP), molecular function (MF) and cellular component (CC) enrichment analyses to further explore the functions of the DEGs in sugar metabolism and ascorbic acid production in the two apricot varieties (Figure 4).

The BP terms cellular processes and metabolic processes and the MF terms cellular anatomical entities and protein‐containing complexes were enriched in the DEGs.

Additionally, the CC terms binding and catalytic activity and the containment complex were enriched in the DEGs. Furthermore, ascorbic acid synthesis was also enriched in the DEGs, which suggests that these genes are involved in the production of soluble sugars and ascorbic acid in these two apricot varieties.

KEGG pathway enrichment analysis revealed that pathways involving ribosomes and motor proteins, the nucleotide sugar biosynthesis pathway, amino sugar and nucleotide sugar metabolic pathways, and α‐linolenic acid metabolic pathways were significantly enriched in the DEGs between the two apricot varieties in X2. K‐means analysis revealed that the DEGs in X2 that were involved in ascorbic acid synthesis exhibited similar expression trends, while the DEGs involved in sugar synthesis exhibited opposite expression trends. Ascorbic acid and alternate metabolism were also enriched in the DEGs in L6, along with other pathways such as those involving nucleosomes and motor proteins and those associated with glycosaminoglycan breakdown, monobasic monomer production, lysine biosynthesis, and purine metabolism. Ascorbic acid and alternative metabolism were also enriched in the DEGs in L2. A correlation study can be performed to determine whether there is a relationship between the two pathways.

Propionic acid metabolism, homologous recombination, and DNA recombination were enriched in the DEGs in the remaining stages. Only glycerophospholipid, selenium compound, and purine metabolism were enriched in the DEGs in X6. The MAPK signaling pathway, phytohormone signaling, carbon metabolism, the synthesis of secondary metabolites, the metabolism of fatty acids, the metabolism of alanine, aspartate, and glutamate, and the breakdown of acetylleucine and oleic acid were all enriched in the DEGs in L1.

### 3.5 Identification of DEGs Associated With Gluconeogenesis and Ascorbic Acid Synthesis Pathways

**FIGURE 5:**
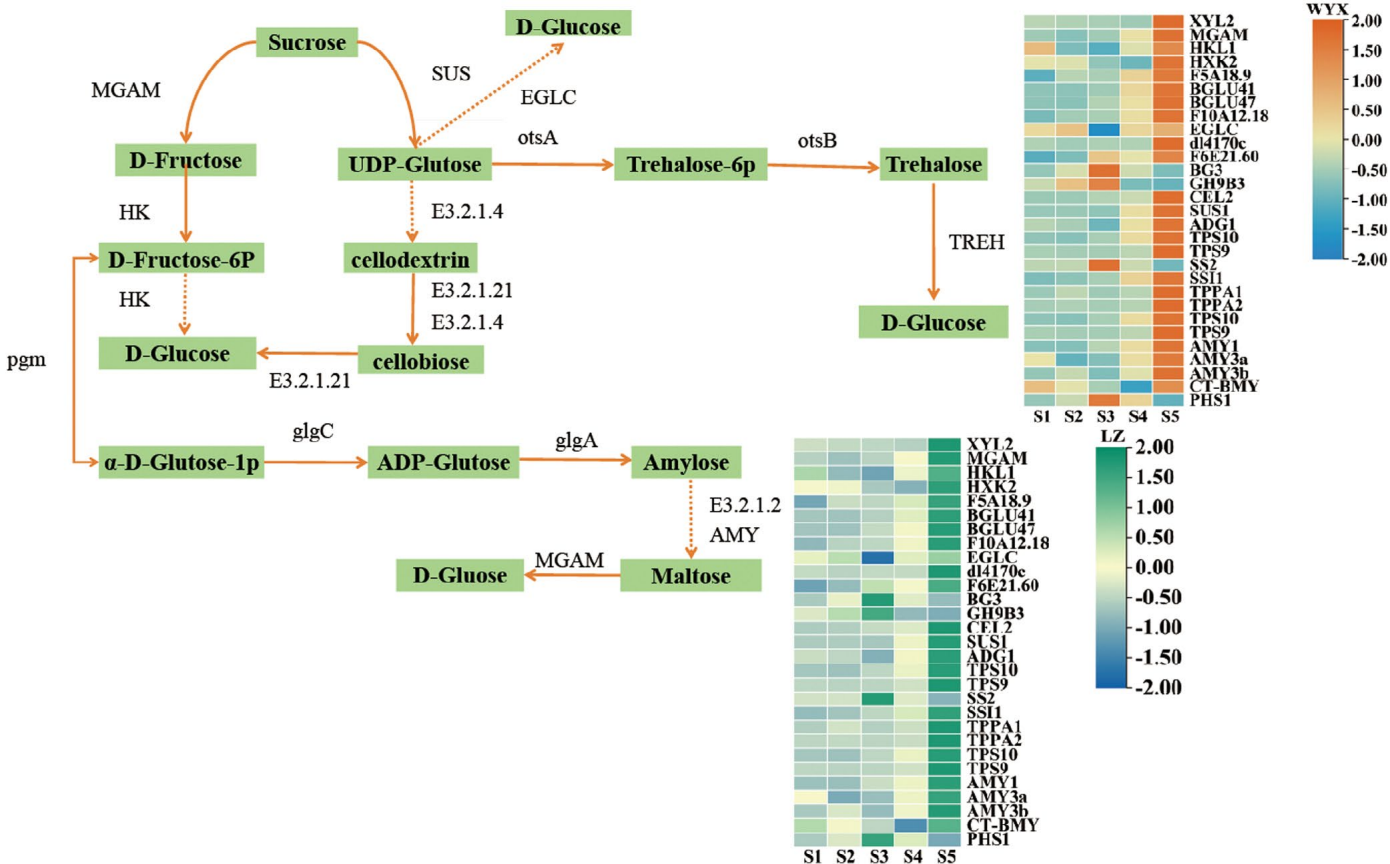
Expression patterns of DEGs involved in sugar metabolism.

DEGs related to the starch and sucrose synthesis were screened to determine the major genes that regulate sugar metabolism (Figure 5). Variations in the expression of these genes led to variations in the sugar content in the apricots in different developmental periods. We verified that the DEGs were involved in the synthesis of sugar by conducting a K‐means analysis, which revealed that their patterns of change matched those of fructose and sucrose.

‘WYX’ expressed 12 unique DEGs involved in the regulation of enzymes in the starch, sucrose, fructose, and glucose metabolic pathways, including *MGAM* (sucrose, PaF106G0400017358.01), *HK* (sucrose‐6‐phosphatase, PaF106G0700026522.01), *TGG2*, *BGLU47* (cellulose, PaF106G0400015877.01, PaF106G0600022723.01), *EGLC* (glucanendo‐1,3‐β‐D‐glucosidase, PaF106G0400015157.01), *GH9B3* (endo‐glucanase, the PaF106G0800032867.01), *PGM* (phospho‐glucose translocase, PaF106G0100003912.01), *otsB* (alginate 6‐phosphate esterase, PaF106G0400018078.01, PaF106G0200010293.01. PaF106G0100001132.01), and *AMY* (β‐amylase, PaF106G0100003246.01, PaF106G0300011580.01). ‘LZ’ expressed four unique DEGs including maltase‐glucoamylase (PaF106G0700028769.01), dextran endo‐1,3‐β‐D‐glucosidase (PaF106G0600022982.01), sucrose synthase (PaF106G0600022982.01), and glucose‐1‐phosphate adenylyltransferase (PaF106G0700026815.01). Moreover, ‘WYX’ and ‘LZ’ exhibited high expression levels of *SUS* (sucrose synthase, PaF106G0700026815.01). These results imply that DEGs linked to sugar metabolism are crucial in controlling sugar accumulation in apricots.

**FIGURE 6:**
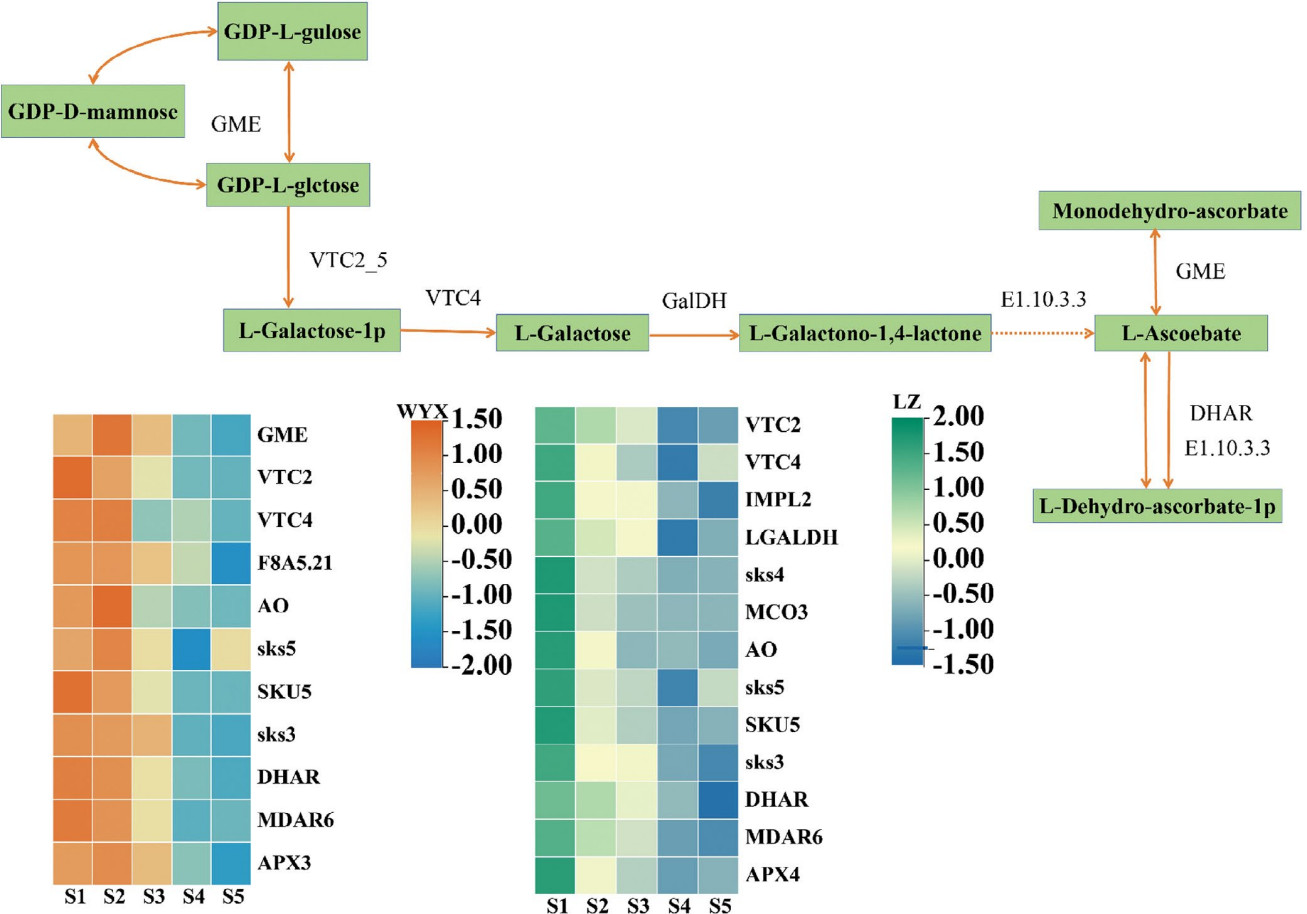
Expression patterns of DEGs involved in ascorbic acid metabolism.

One of the key components of high‐quality apricots is ascorbic acid (Figure 6). The DEGs involved in the ascorbic acid synthesis pathway were analyzed, with notable variations in expression occurring in different growth periods. These genes included *GMEs* (GDP‐D‐mannose 3′,5′‐differential anisomerase, in ‘WYX’ PaF106G0100003725.01), *VTC2_5* (GDP‐L‐galactose phosphorylase, PaF106G0200008910.01), *VTC4* (inositol phosphatase/L‐galactose 1‐phosphate phosphatase, PaF106G0400018346.01), *GalDH* (L‐galactose dehydrogenase, PaF106G0100000284.01), *AO*, *SKS5*, *SKU5*, SKS*3* (L‐ascorbic acid oxidase, PaF106G0400016721.01, PaF106G0100004207.01, PaF106G0500019378.01, and PaF106G0800030285.01), *DHAR* (glutathione dehydrogenase/transferase, PaF106G0200009695.01), *MDAR6* (monodehydroascorbic acid reductase (NADH), PaF106G0500019749.01), and *APX3* (L‐ ascorbic acid peroxidase, PaF106G0700027091.01). Several genes differed in expression between ‘LZ’ and ‘WYX’, and these genes were *VTC4* (inositol phosphatase/L‐galactose 1‐phosphate phosphatase, PaF106G0400018346.01, PaF106G0800031491.01), *GalDH* (L‐galactose dehydrogenase, PaF106G0800030702.01), *SKS4*, *SKS5* (L‐ascorbic acid oxidase, PaF106G0400018234.01, PaF106G0300012038.01), and *APX4* (L‐ascorbic acid peroxidase, PaF106G0100006021.01). In addition, *SKS5*, *SKU5* (L‐ascorbic acid oxidase, PaF106G0100004207.01, PaF106G0500019378.01), a downstream metabolite of ascorbic acid, was highly expressed in ‘WYX’ and ‘LZ’.

### 3.6 Identification of Coexpression Networks and Hub Genes Related to Sugar Metabolism and Ascorbic Acid Synthesis

**FIGURE 7:**
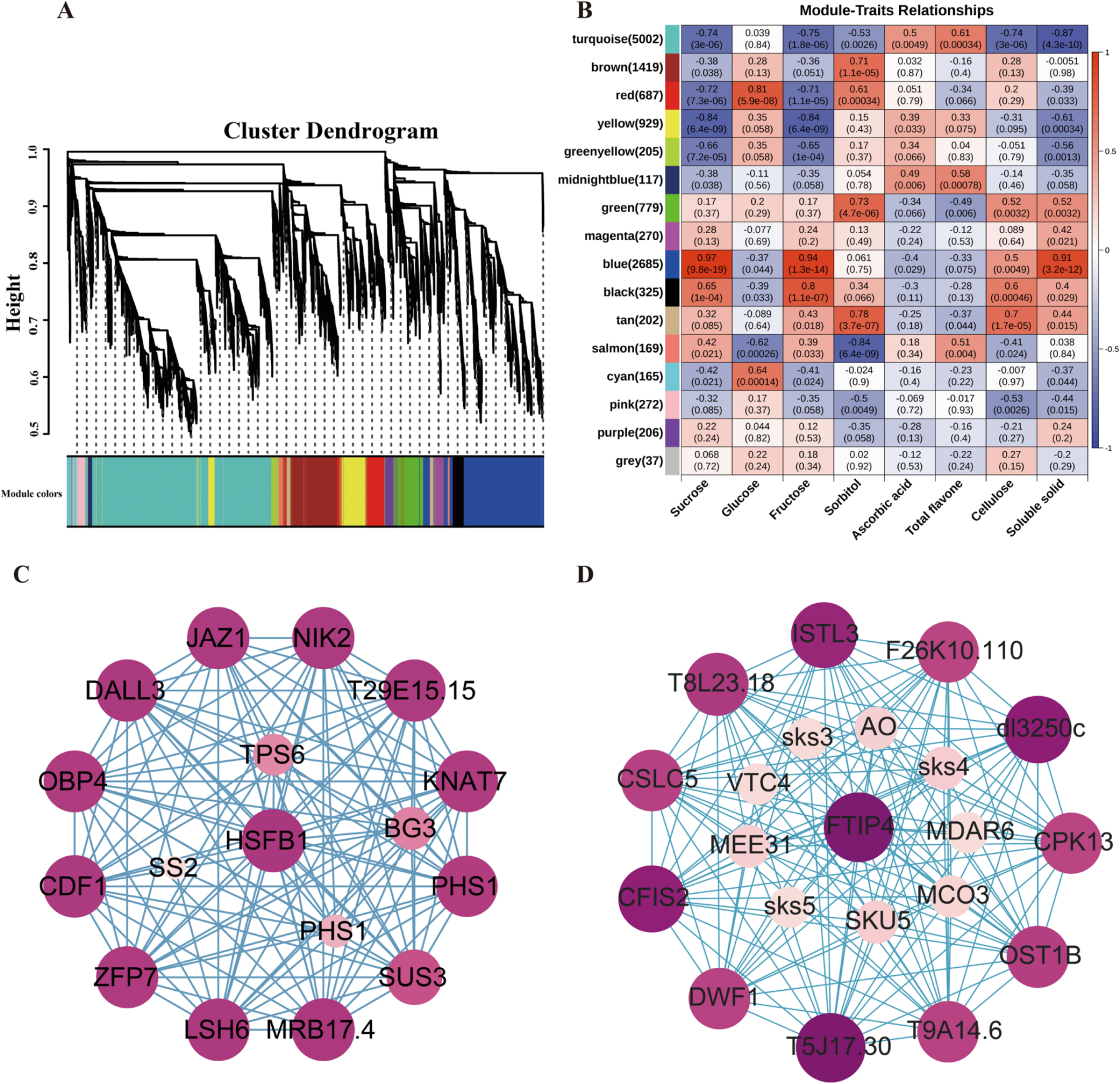
Identification of hub genes in the coexpression network. (a) WGCNA hierarchical clustering tree of 16 modules of coexpressed genes (clustering dendrogram). Some genes always have similar expression changes in a physiological process or different tissues. These genes may be functionally related, and they can be defined as a module (module). For the upper half of the dendrogram, the longitudinal distances represent the distance between two nodes (between genes), and the horizontal distance is meaningless. (b) Heatmap of sugar and ascorbic acid contents; each row represents a module with different colors to indicate correlation values from −1 to 1. (c) Blue module coexpression network module (d) Turquoise module coexpression network module.

We examined the gene regulatory networks linked to the synthesis of sucrose, glucose, fructose, and ascorbic acid and their expression patterns via WGCNA. Following DEG identification, 13,469 genes were analyzed via transcriptome sequencing, and the genes were further split into 16 expression modules (Figure 7a). The findings indicated (Figure 7b) that the red module had the strongest correlation with glucose synthesis (*r* = 0.81, *p* = 5.9e^−8^), the blue module (2685) had the strongest correlation with fructose synthesis (*r* = 0.94, *p* = 1.3e^−14^) and sucrose synthesis (*r* = 0.97, *p* = 9.8e^−19^), and the turquoise (5002) module had the strongest correlation with ascorbic acid synthesis (*r* = 0, *p* = 0.0049). Interestingly, the expression of genes in the blue module decreased throughout the developmental stages and was significantly expressed in XE1, XE2, XE3, LE1, LE2, and LE3. Both the blue and turquoise modules followed the same expression trend. On the basis of high gene significance (GS) and high module affiliation (MM), we screened the genes in the blue module.

With a total of 20 genes (Figure 7c), the gene coexpression network was built on the basis of the weighted values and the degree of linkage between the candidate genes and the DEGs involved in glucose metabolism. Notably, *HSFB1* was identified as a key hub gene with 18 linkage edges, and 6 structural genes related to glucose metabolism had the highest values of the 14 top‐ranked genes. *FTIP4* was shown to be the primary hub gene in the turquoise module (Figure 7d). To construct the gene coexpression network, we chose the top 12 genes with the highest degree values and the top nine DEGs involved in ascorbic acid production.

### 3.7 qRT–PCR Analysis

**FIGURE 8:**
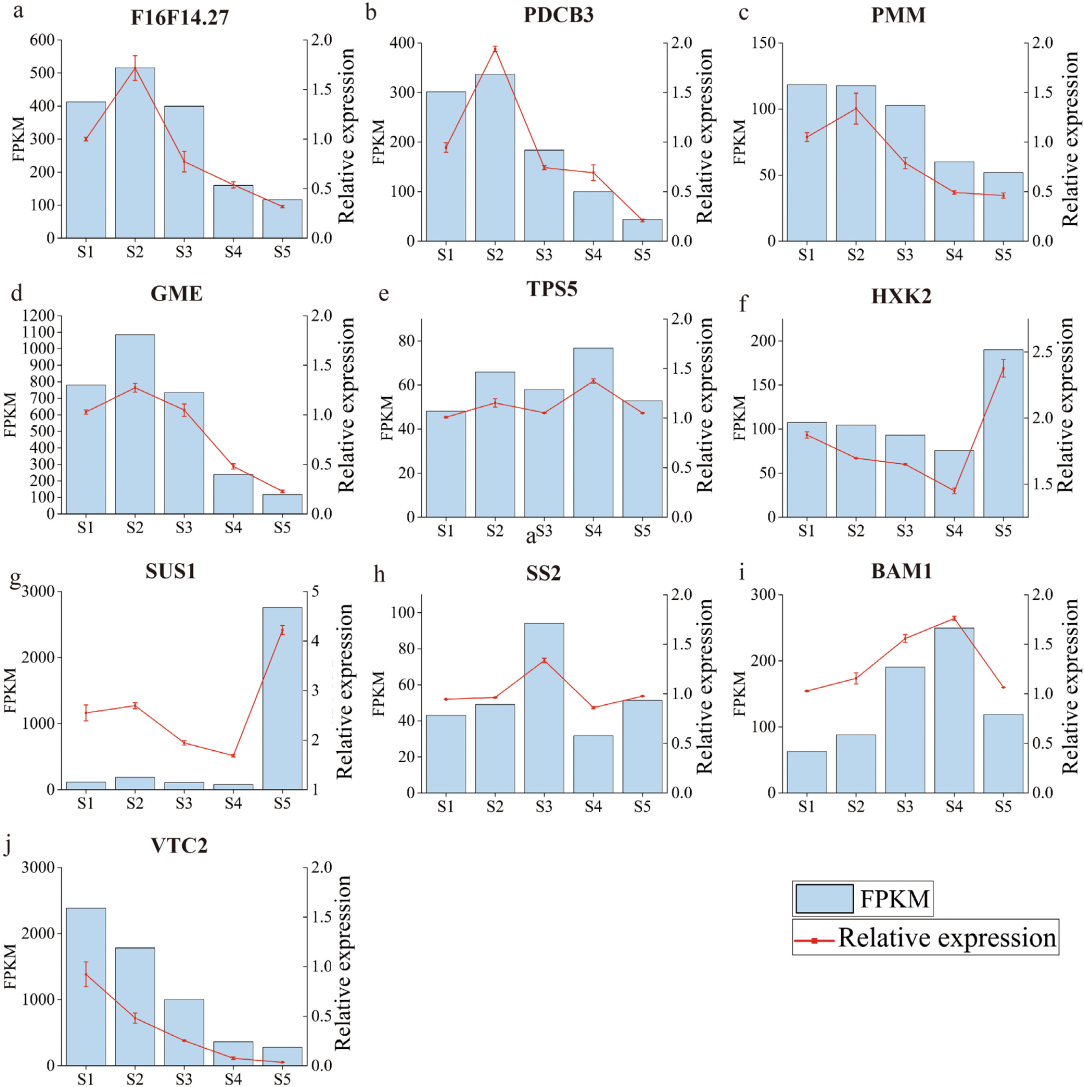
The expression levels of 10 candidate DEGs were verified via qRT–PCR. The columns and lines represent the RNA‐seq and qRT–PCR data, respectively, of the candidate DEGs. 18S rRNA was used as the internal control. (a) F16F14.27, (b) PDCB3, (c) PMM, (d) GME, (e) TPS5, (f) HXK2, (g) SUS1, (h) SS2, (i) BAM1, (j) VTC2. The error bars indicate the SDs.

The relative expression of 11 candidate genes linked to the pathways involved in ascorbic acid synthesis and sugar metabolism in the two apricot varieties was confirmed via qRT–PCR to assess the accuracy of the RNA‐seq results (Figure 8). The qRT–PCR results demonstrated a steady expression trend, which was consistent with the RNA‐seq results. These results show the accuracy of the transcriptome analysis and the reliability of the data.

## 4 Discussion

In apricots, sugar is a key metabolite that plays a critical role in maintaining proper fruit growth and development, facilitating the synthesis of nutrients, and promoting the accumulation, including fructose, glucose, and sucrose (Alajil et al. 2021; Tian et al. 2023). We examined sugar accumulation in apricots during five developmental stages (the young fruit stage, rapid growth stage, nodule stage, color change stage, and commercial ripening stage). Under the combined action of photosynthesis and sugar metabolism in plant bodies, the contents of sucrose, glucose, fructose, and sorbitol dynamically change and are the main components of soluble sugars in Rosaceae.

During the developmental stages, sucrose was the dominant sugar accumulated in both ‘WYX’ and ‘LZ’. The sugar accumulation in ripe fruits was 8 and 6 times higher in ripe fruits, respectively, which was consistent with the sucrose content findings in previous studies (Gou et al. 2023). At the color change and ripening stages, ‘WYX’ had higher sucrose and glucose contents than ‘LZ’ did, and from the fast‐growing stage onwards, ‘LZ’ had a significantly higher sorbitol content than ‘WYX’. From the fast‐growing stage onwards, the sorbitol content of ‘LZ’ was significantly greater than that of ‘WYX’. Since the sorbitol content increases with altitude (Sarıdaş et al. 2024), this may be due to ‘WYX’ being a native species of Hohhot, at an altitude of 1040 m above sea level, whereas ‘LZ’ is an imported species from Lanzhou city, Gansu Province, growing at an altitude of 1520 m above sea level. The variations in soluble sugar contents between the two apricot varieties may be attributed to differences in their growing locations. Glucose was the predominant sugar present during the early stages of fruit growth and development. However, with fruit maturation, glucose level decreased, while sucrose and sorbitol contents increased. The activities of sucrose synthase and succinate dehydrogenase are linked to the shift in sugar metabolism from catabolism to anabolism. Consequently, the total sugar and sucrose contents of the fruit increased (Chen et al. 2009; Zheng et al. 2016; Weng 2017).

During the experiment, we discovered that the change in the AsA content of ‘WYX’ as inversely related to the sugar content. This could be due to the pH of the fruit, as pH has a significant impact on the stability of ascorbic acid. Additionally, a decrease in sugar content may be accompanied by an increase in pH, which could have an indirect effect on ascorbic acid metabolism (Liu and Xu 2015). Furthermore, as a potent antioxidant, ascorbic acid may exacerbate oxidative stress in response to increased fruit sugar buildup, necessitating increased ascorbic acid intake to preserve redox balance. However, if the antioxidant system is robust enough, the need for ascorbic acid may decline during ripening, which would result in a decrease in its level. Moreover, ethylene, a plant hormone essential for fruit ripening, may influence ascorbic acid metabolism and sugar accumulation. Ethylene may hinder the synthesis of ascorbic acid and promote sugar accumulation. To understand the precise link between sugar production and ascorbic acid metabolism, extensive experimental research is needed.

In this study, 15 different sugar transporter genes, including *MGAM*, *HK*, *TGG2*, *BGLU47*, *EGLC*, *GH9B3*, *PGM*, *otsA*, *otsB*, *AMY*, *SUS*, *glgA*, *glgC*, and *PYG* were detected. Among these genes, *HK* (PaF106G0700026522.01), *EGLC* (PaF106G0600022982.01), *SUS* (PaF106G0700026815.01), *PGM* (PaF106G0100003912.01), and *glgC* (PaF106G0300012278.01) significantly influenced fruit sweetness. Among these, *SUS* (PaF106G0700026815.01) was highly expressed in both apricot varieties. Previous research (Aslam et al. 2019) also indicated that the SUS gene plays a critical role in sugar the accumulation and sweetness in apricots. In the metabolism of plants and animals, ascorbic acid is a essential antioxidant molecule and an enzyme cofactor (Fenech et al. 2018). The consumption of plants is a primary means through which humans can obtain their recommended daily intake of ascorbic acid. As ascorbic acid can be synthesized in fruiting plants via a variety of pathways, its biosynthesis is differentially regulated by the expression of important genes. Producing transgenic fruits and vegetables that are high in ascorbic acid can be achieved by better understanding of ascorbic acid anabolism as well as the role and expression of associated genes in fruiting plants. The growth trend of ‘WYX’ initially showed an increase, followed by a decrease, mirroring the trend observed in the AsA levels of two different types of kiwi fruit. Similarly, ‘LZ’ and peach followed comparable trends, consistent with findings from previous studies (Bulley et al. 2009; Shu et al. 2023; Imai et al. 2009). The AsA concentration of ‘LZ’ was three times greater than that of ‘WYX’ during the S1 period and gradually decreased over the ripening stage (Ioannidi et al. 2009). However, this pattern didnot occur in tomatoes. During the S2 (fast‐growing) period, ‘WYX’ had the maximum amount of ascorbic acid, before gradually decreased. During the same period, the level of ascorbic acid in ‘LZ’ was higher than during ripening. GalLDH catalyzes the oxidation of the final substrate, L‐GalL, to AsA via the main l‐galactose or Smirnoff–Wheeler pathway for AsA biosynthesis in plant cells. It is also essential d‐galacturonide pathway (Hancock and Viola 2005), where GalDH is the critical enzyme that determines whether the plant synthesizes AsA via the L‐galactose pathway. Furthermore, young fruits have a greater capacity to convert GalL to AsA (Li et al. 2008), leading to higher AsA concentration during the S2 stage. Since AsA accumulation is mainly determined by the balance between DHA synthesis and oxidative loss in plant cells, the AsA content of both fruits began to decrease from S2 onwards. This occurred because the rate of AsA synthesis in young fruit was lower than the rate of oxidative loss during fruit development (Hancock and Viola 2005). The genome of *T acutus* contains genes that encoding glucose‐phosphate isomerase (*PGI1*), mannose‐6‐phosphate isomerase (*PMI1*), phosphoadenosine mutase (*PMM*), mannose pyrophosphorylase (*GMP1*), GDP mannose‐3′, 5′ epimase (*GME1* and *GME2*), involved in the biosynthesis and accumulation of ascorbic acid via the L‐galactose pathway in immature fruits (Dos Santos et al. 2019). Four linear and cyclic pathways, involving numerous enzymes directly or indirectly involved in the metabolic reactions of AsA, regulate the synthesis of AsA (Davey et al. 2000; Wolucka and Van Montagu 2003; Wheeler et al. 1998). In this study, 10 regulators of AsA synthesis were identified: *GME*, *VTC2_5*, *VTC4*, *DHAR*, *MDAR6*, *GalDH*, *APX4*, *SKS4*, and *SKS5*. We deduced that APX4, SKS4, and SKS5 (L‐ascorbic acid oxidase, PaF106G0100004207.01, PaF106G0500019378.01), highly expressed in both varieties, may be crucial enzymes in the regulation of AsA synthesis. Different AsA synthesis routes and accumulation patterns in plants. For example, the l‐galactose and d‐galacturonic acid pathways exist in strawberries, and they each have different functions in maintaining AsA levels in immature and ripe strawberries (Cruz‐Rus et al. 2011). In kiwifruit, the expression of the L‐glucuronolactone oxidase gene is strongly correlated with ascorbic acid content, the balance between AsA synthesis and catabolism is tightly regulated, and environmental factors regulate the metabolic pathway by influencing gene transcription (Dos Santos et al. 2019; Wang et al. 2023).

WGCNA revealed complex systemic gene relationships. We identified gene modules characterized by strong coexpression associated with the metabolism of sucrose and ascorbic acid. By analyzing the relationships between these modules and various physiological markers, we inferred their roles in metabolic regulation. The blue and turquoise modules were derived from WGCNA of the K‐means clustering results on the basis of sugar and ascorbic acid contents. *HSFB1* and *FTIP4* emerged as the top‐ranked hub genes based on their degree values. The top four hub genes (*dl3250c*, *T5J17.30*, *ISTL3*, *CFIS2*) and the top 11 hub genes (*MRB17.4*, *OBP4*, *DALL3*, *LSH6*, *T1B9.2*, *CDFI*, *JAZ1*, *CIA2*, *NIK2*, *T29E15.15*, *ZFP7*, *KNAT7*) were analyzed in coexpression studies with the DEGs involved in metabolic pathways. Gene coexpression analyses revealed that seven DEGs were associated with hub genes involved in gluconeogenesis and ascorbic acid synthesis.

The role of *HSFB1* in sugar metabolism differed from that of *HSFB1* in sugar content. The production of a trait often involves the joint expression of multiple genes. Research has shown that the inhibition or expression of *HSFB1* can increase plant tolerance to heat. For example, in Arabidopsis, *HSFB1* represses transcription and negatively regulates the expression of heat‐induced stress resistance genes (Ikeda et al. 2011); in tomato leaves, *HSFB1* knocked out improves heat tolerance (Paupière et al. 2020); and in grapes, *HSFB1* was found to be a key regulator of heat tolerance (Ye et al. 2020). Plant heat tolerance has a positive effect on the accumulation of sucrose and glucose (Commisso et al. 2016; Liu et al. 2019).

We discovered that *FTIP4*, a key regulator of AsA synthesis, plays a crucial role in the regulation of AsA synthesis, as evidenced by its consistent correlation with AsA content. *FTIP3/4* is a crucial regulatory factor controlling plant cell differentiation and tissue formation by influencing the intracellular transportation of the SHOOT MERISTEMLESS (STM) protein (Liu, Li, et al. 2018). The STM protein operates on the plasma membrane of plant cells, facilitating the synthesis of cytokinin (CK) to prevent premature cell differentiation and assisting undifferentiated cells in the formation of self‐sustaining meristematic tissue, which is vital for plant growth and development (Scofield et al. 2014). In strawberry research, spraying cytokinins like 6‐benzylaminopurine (6‐BA) and kinetin (KT) effectively increased the vitamin C content in strawberries. These regulators affect the internal hormonal balance of the plant and promote the biosynthesis of vitamin C (Yuan 2019). Recent studies on the *FTIP* gene family indicate that *FTIP1* is involved in the molecular mechanisms underlying the response of rice to drought stress, which has great implications for understanding the genetic basis of plant drought resistance (Chen et al. 2021). Given that genes within the same family may exhibit functional similarities (Liu, Liu, and Rajapakse 2018) and considering the role of ascorbic acid as a potent antioxidant, *FTIP1* may aid plants in mitigating reactive oxygen species generated under drought conditions, thereby protecting cells from oxidative damage and enhancing drought resistance (Davey et al. 2000; Mellidou and Kanellis 2017; Fenech et al. 2018). Based on the identified hub genes, we hypothesize that *FTIP4* may be involved in the synthesis of ascorbic acid.

These studies included metabolite analysis, gene expression analyses, and physiological and biochemical tests providing insights into ‘the optimization of fruit quality and nutrition’.

## 5 Conclusions

We utilized transcriptome sequencing to investigate the gene expression associated with apricot fruit development and analyzed sugar and ascorbic acid levels in ‘WYX’ and ‘LZ’. We identified sugar transport genes, ascorbic acid synthesis regulatory factors, and content differences. At maturity, ‘WYX’ accumulated more sucrose, whereas ‘LZ’ accumulated more sorbitol. In the early fruit stage, ‘LZ’ had a relatively high ascorbic acid content, which decreased as the fruit aged. Our identification of the genes *FTIP4* and *HSFB1* provides new insights into apricot fruit metabolism. However, importantly, transcriptome analysis represents only a fraction of metabolic regulation; further investigation is needed to identify other mechanisms. This study contributes to our understanding of apricot metabolism and aids in the establishment of germplasm resources and molecular breeding for improved apricot varieties.

## Author Contributions

**Yanhong He:** conceptualization (equal), data curation (equal), formal analysis (equal), funding acquisition (equal), investigation (equal), methodology (equal), project administration (equal), resources (equal), software (equal), supervision (equal), validation (equal), visualization (equal), writing – original draft (equal), writing – review and editing (equal). **Yang Zhao:** conceptualization (equal), data curation (equal), formal analysis (equal), funding acquisition (equal), investigation (equal), methodology (equal), project administration (equal), resources (equal), software (equal), supervision (equal), validation (equal), visualization (equal), writing – original draft (equal), writing – review and editing (equal). **Tianxing Guo:** resources (supporting). **Wenbao Jia:** software (supporting). **Chun Tian:** supervision (supporting). **Yu‐e Bai:** conceptualization (lead).

## Ethics Statement

The landowner allows access to the orchard for the purpose of collecting fruit.

## Consent

The authors have nothing to report.

## Conflicts of Interest

The authors declare no conflicts of interest.

## Data Availability

All data and materials are presented in the main paper and additional Supporting Information. All raw sequence data are available on the National Center for Biotechnology Information (NCBI) website with the SRA accession number of PRJNA11653489 (http://www.ncbi.nlm.nih.gov/bioproject/1165348).
